# ABA and the ubiquitin E3 ligase KEEP ON GOING affect proteolysis of the *Arabidopsis thaliana* transcription factors ABF1 and ABF3

**DOI:** 10.1111/tpj.12259

**Published:** 2013-06-06

**Authors:** Yi-Tze Chen, Hongxia Liu, Sophia Stone, Judy Callis

**Affiliations:** 1 Department of Molecular and Cellular Biology, UC-Davis1 Shields Ave, Davis, CA, 95616, USA; 2 Plant Biology Graduate Group, UC-Davis1 Shields Ave, Davis, CA, 95616, USA; 3 Department of Biology, Dalhousie University1355 Oxford Street, Halifax, NS, B3H 4J1, Canada

**Keywords:** *Arabidopsis thaliana*, ABF, abscisic acid, ubiquitin E3 ligase, ubiquitin, proteolysis, KEG

## Abstract

The ABA Binding Factor/ABA-Responsive Element Binding Proteins (ABF/AREB) subfamily of bZIP-type transcription factors are positive effectors of ABA responses. Here, we examine the proteolytic regulation of two members: *Arabidopsis thaliana* ABF1 and ABF3. Both transcription factors are unstable in seedlings, and their degradation is sensitive to proteasome inhibition. ABA treatment of seedlings leads to their rapid accumulation, the result of slowed proteolysis. Deletion of the conserved C–terminal region required for 14–3–3 interaction destabilizes the proteins. The degradation of ABF1 and ABF3 are slower *in vivo* in seedlings lacking the ubiquitin E3 ligase KEEP ON GOING (KEG), and *in vitro* in extracts from *keg* seedlings, implicating KEG in their degradation. ABF1 and ABF3 are ubiquitylation substrates of KEG *in vitro*, and *in vitro* pull-down assays document their direct interaction. In contrast to ABI5, another KEG substrate, the degradation of ABFs and proteolytic regulation of ABFs by ABA still occurs in *keg* seedlings, suggesting that additional E3s participate in ABF1 and ABF3 proteolysis. Loss of ABF1 or ABF3 in the *keg* background has a phenotypic effect similar to the loss of ABI5, and there is no additional rescue of the *keg* phenotype in *abf1 abf3 abi5 keg* seedlings. This result suggests that the abundance of other substrates is altered in *keg* seedlings, affecting growth. In conclusion, ABF1 and ABF3 abundance is affected by ABA and KEG, and the conserved C4 region serves as a stabilizing element.

## Introduction

Abscisic acid (ABA) is a phytohormone that affects many important aspects of plant development, including seed maturation, seed desiccation and dormancy, transition from the seed to seedling stage, and flowering and fruit ripening. ABA also mediates plants' responses to abiotic stresses such as drought, salinity, and cold temperature (Finkelstein *et al*., 2002; Qin *et al*., 2011). ABA signal transduction, from perception of environmental cues to physiological responses, involves many components, including ABA receptors, protein kinases, phosphatases, transcription factors, and ABA-induced genes containing conserved G–box-like *cis*-acting elements (ABREs) in their promoter regions (Yoshida *et al*., 2010; Fujita *et al*., 2011, 2013). During germination and post-germinative growth, the bZIP transcription factor, ABI5 (ABSCISIC ACID-INSENSITIVE 5) plays an important role, and increased ABI5 is associated with delayed growth (Kim *et al*., 2002; Lopez-Molina *et al*., 2002).

ABI5 is a member of a subfamily of bZIP transcription factors (Jakoby *et al*., 2002). In addition to ABI5, seven other ABI5-related transcription factors, named AREBs (ABA-Responsive Element Binding Proteins), ABFs (ABRE Binding Factors) and/or DPBFs (Dc3 Promoter Binding Factors) have been functionally characterized as components in ABA signaling (Jakoby *et al*., 2002; Fujita *et al*., 2011). The basic region of the bZIP domain is highly conserved among these subfamily members. In addition to the bZIP region, there are four additional conserved domains, three (C1, C2, and C3) in the N–terminal half and one (C4) at the C–terminus. Within C1, C2, C3 and C4 domains are well-conserved consensus phosphorylation sites for protein kinases.

Several members appear to function redundantly with ABI5. ABI5, ABF1 and ABF3 are able to interact with ABI3, a transcription factor involved in seed maturation and dormancy, and ABF3 has been shown to have redundant functions with ABI5 (Finkelstein *et al*., 2005). Although an *abf3* mutant does not show an increased seed germination rate, it shows a strong ABA-resistant root growth phenotype compared with the wild type. In addition, an *abf3 abi5* double mutant has enhanced ABA resistance in germination and root growth assays, relative to *abf3* and *abi5* single mutants, indicating that ABI5 and ABF3 act redundantly but distinctly in ABA-mediated seed germination and seedling development (Finkelstein *et al*., 2005).

The abundance of ABI5 is affected by transcriptional and post-translational mechanisms (Lopez-Molina *et al*., 2001). *ABI5* mRNAs are increased upon ABA application, and ABI5 protein degradation is slowed in the presence of high levels of ABA, probably through the ubiquitin pathway, leading to a large increase in protein (Lopez-Molina and Chua, 2000; Lopez-Molina *et al*., 2001; Finkelstein *et al*., 2005). The ubiquitin RING-type E3 ligase KEEP ON GOING (KEG) affects ABI5 levels *in vivo* in seedlings, and ubiquitylates ABI5 *in vitro*. Loss of *KEG* results in ABI5 protein hyperaccumulation and ABA hypersensitivity (Stone *et al*., 2006; Liu and Stone, 2010); however, loss of *ABI5* in the *keg* mutant background can only partially rescue the *keg* phenotype. This leaves open the possibility that the abundance of other bZIP transcription factors could also be affected by KEG.

To understand the control of abiotic stress responses, we investigated the proteolytic regulation of two ABI5-related transcription factors: *Arabidopsis thaliana* ABF1 and ABF3. We show that the abundance of ABF1 and ABF3 proteins is affected by ABA and the ubiquitin pathway. The loss of *KEG* affects ABF1 and ABF3 degradation *in vivo* and *in vitro*, and KEG ubiquitylates them *in vitro*; however, ABF1 and ABF3 levels appear to be affected additionally by another, not yet identified, E3. Loss of *ABF1* or *ABF3*, or both, in the *keg* and *keg abi5* mutant backgrounds only partially rescues the *keg* phenotype, suggesting that KEG has additional substrates that have roles in seedling growth.

## Results

### Degradation of ABI5-related transcription factors ABF1 and ABF3 can be detected *in vivo*, and is slowed by proteasomal inhibition

**Figure 1:**
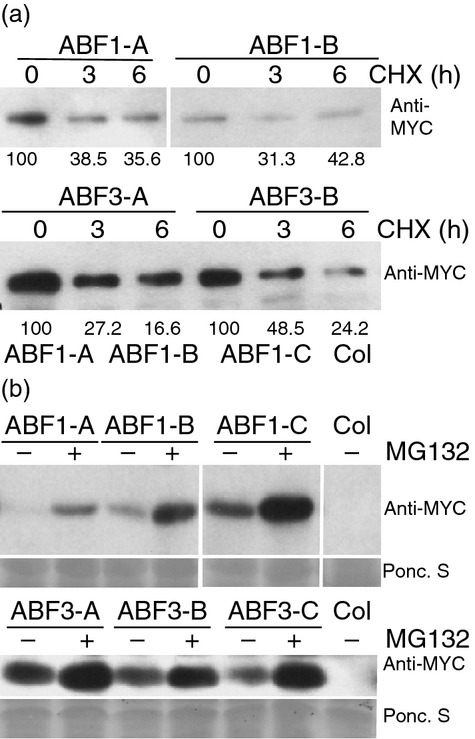
Degradation of ABF1 and ABF3 can be detected by CHX chase assay, and their levels are sensitive to MG132.(a) Seedlings from independent transgenic lines (designated A–C) expressing Myc-ABF1 (top) or Myc-ABF3 (bottom) under the control of the *35S* promoter were grown for 6 days and treated with CHX for 3 or 6 h. CHX 0 represents mock treatment with solvent alone for 6 h. Proteins were extracted and immunoprecipitated by anti-Myc beads. The results were visualized after SDS-PAGE by anti-Myc immunoblotting. The number below represents signal intensity quantified by ImageJ, normalized to CHX 0.(b) Seedlings grown as described in (a) were treated with MG132 or solvent control for 24 h, then proteins were extracted and 100 μg visualized by SDS-PAGE and anti-Myc immunoblotting. Ponceau S staining was used as the loading control.

To investigate ABI5-related bZIP transcription factors at the protein level, we generated multiple independent *Arabidopsis thaliana* lines expressing Myc-tagged bZIP transcription factors under control of the *35S* promoter. Cycloheximide (CHX) chase assays were employed to determine whether protein degradation could be detected. Seedlings from independent lines expressing Myc-ABF1 (Figure 1a, top) or Myc-ABF3 (Figure 1a, bottom) were treated with CHX or buffer over a period of 6 h. Although there is some variability between experiments, there is a consistent loss of protein after CHX treatment. Including all lines and replicas, an average of 29 ± 15% (SD, *n* = 9) and 23 ± 14% (SD, *n* = 6) of Myc-ABF1 and Myc-ABF3, respectively, remained after 3 h of treatment with CHX.

We next tested whether the observed degradation requires the proteasome, which provides the major proteolytic activity in cells. Seedlings expressing Myc-ABF1 or Myc-ABF3 were treated with the proteasome inhibitor MG132 (Figure 1b). Both Myc-ABF1 and Myc-ABF3 proteins accumulated in MG132-treated seedlings, suggesting that slowed degradation through proteasomal inhibition led to increases in Myc-ABF1 and Myc-ABF3 levels.

### ABF1 and ABF3 accumulate in response to ABA because of slowed degradation

**Figure 2:**
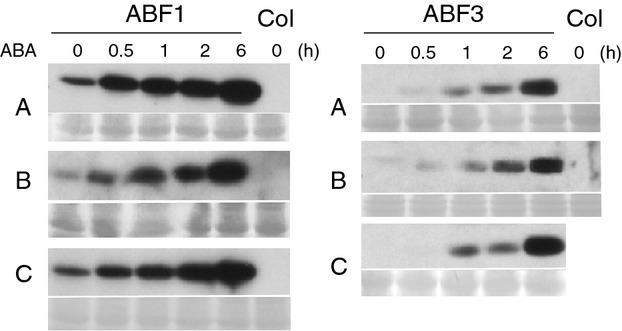
ABF1 and ABF3 proteins rapidly accumulate in response to ABA.Seedlings from three independent transgenic lines expressing either Myc-ABF1 (left) or Myc-ABF3 (right) were grown for 6 days and treated with ABA for 0.5, 1, 2 or 6 h. Time 0 represents mock treatment with solvent alone for the length of 6 h. Proteins were extracted and the results were visualized after SDS-PAGE and anti-Myc immunoblotting. Ponceau S staining was used as the loading control. Col represents non-transgenic control seedlings.

Previous studies showed that ABA enhances ABI5 protein stability (Lopez-Molina *et al*., 2001). ABF3 also accumulates in response to ABA treatment in mature leaves (Sirichandra *et al*., 2010). To examine whether ABF1 or ABF3 protein degradation is affected in response to ABA in young seedlings, we first tested whether ABA affects their protein levels. Seedlings expressing Myc-ABF1 or Myc-ABF3 were treated with ABA or solvent over a 6–h time course (Figure 2). Both Myc-ABF1 and Myc-ABF3 started to accumulate by 30 min, and continued to accumulate through the 6–h period. Because expression is under control of the *35S* promoter, a promoter not responsive to ABA treatment (Mundy *et al*., 1990; Gampala *et al*., 2002; Zhang *et al*., 2007; Xi *et al*., 2010; Wang, 2013), the observed protein accumulation by ABA should be post-transcriptional. As expected, in all lines, *Myc-ABF1* and *Myc-ABF3* mRNAs with or without 6 h of ABA, as measured by qPCR, were not statistically different (Figure S1).

**Figure 3:**
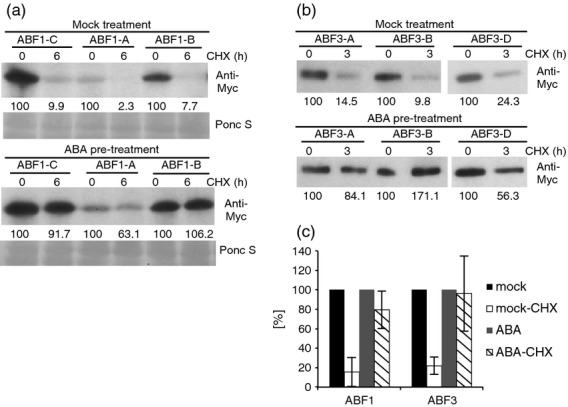
ABA slows down ABF1 and ABF3 protein degradation.Seedlings from independent transgenic lines expressing Myc-ABF1 (a) or Myc-ABF3 (b) were grown for 6 days and treated with ABA or with ethanol as mock treatment for 6 h, then treated with CHX for the indicated times. Proteins were extracted (a), or were extracted and immunoprecipitated by anti-Myc beads (b). Results were visualized after SDS-PAGE by anti-Myc immunoblotting. The percentages represent signal intensities quantified by ImageJ normalized to CHX 0. Ponceau S staining was used as the loading control.(c) Data from independent experiments were plotted normalized to 0 CHX (ABF1, *n* = 6; ABF3, *n* = 8). The percentage remaining is statistically different in ABA samples than the control by a Student's *t*–test, with *P* < 0.0001 for both ABF1 and ABF3.

To distinguish between translational or post-translational effects, seedlings were treated with ABA or mock-treated for 6 h (pre-treatment), and then CHX was added to measure degradation directly. Without ABA pre-treatment, more than 90% of Myc-ABF1 was degraded after 6 h of treatment with CHX (Figure 3a, upper panels), whereas with ABA pre-treatment, less than 40% of Myc-ABF1 was degraded (Figure 3a, lower panels). Similarly, without ABA pre-treatment, more than 75% of Myc-ABF3 was degraded after 3 h of treatment with CHX (Figure 3b, upper panel), whereas with ABA, less than 50% of Myc-ABF3 was degraded (Figure 3b, lower panel). Combining experiments, the CHX chase assays clearly show that ABA slows Myc-AB1 and Myc-ABF3 degradation rates (Figure 3c).

### ABF1 and ABF3 degradation is slowed in *keg* seedlings

The ubiquitin E3 ligase KEG is implicated in modulating ABI5 protein (Stone *et al*., 2005; Liu and Stone, 2010). To examine if proteolysis of ABF1 and ABF3 is also affected by KEG, the same transgenes analyzed above were introduced into the *KEG/keg–1* background. If ABFs are KEG ubiquitylation substrates, and this modification targets them for degradation, then the expectation is that they should hyperaccumulate in *keg* seedlings. Consistent with previous studies (Stone *et al*., 2005; Liu and Stone, 2010), endogenous ABI5 hyperaccumulated in *keg* roots compared with wild-type siblings, where no ABI5 signal was detected (Figure S2a, middle panel). In contrast, the opposite was observed for Myc-ABF3 in these genetic backgrounds: the Myc-ABF3 signal was barely detectable in *keg* roots, whereas a strong signal was detected in wild-type roots (Figure S2a, top). Using whole seedlings, Myc-ABF3 was at a slightly higher level in *keg* seedlings compared with wild-type siblings, but this was not strong hyperaccumulation, as observed for ABI5 in the same extracts (Figure S2b). Similarly, in Myc-ABF1 transgenic lines, a stronger signal was detected in *keg* whole seedlings than in wild-type seedlings, indicating that Myc-ABF1 hyperaccumulates in *keg* seedlings but not as dramatically as ABI5 (Figure S2c).

Although Myc-ABF1 and Myc-ABF3 expression is under the control of the *35S* promoter, perhaps Myc-ABF3 does not hyperaccumulate in *keg* compared with wild-type siblings because Myc-ABF3 mRNA is reduced in *keg*. Using qPCR, 17.7-, 8.7- and 7.7–fold more Myc-ABF3 mRNA was detected in wild-type seedlings compared with sibling *keg* seedlings for lines A–C, respectively (Figure S2d). Thus, differences in Myc-ABF3 protein between wild-type and *keg* seedlings are obscured by the unexpected reduction of Myc-ABF3 mRNA in *keg* seedlings.

**Figure 4:**
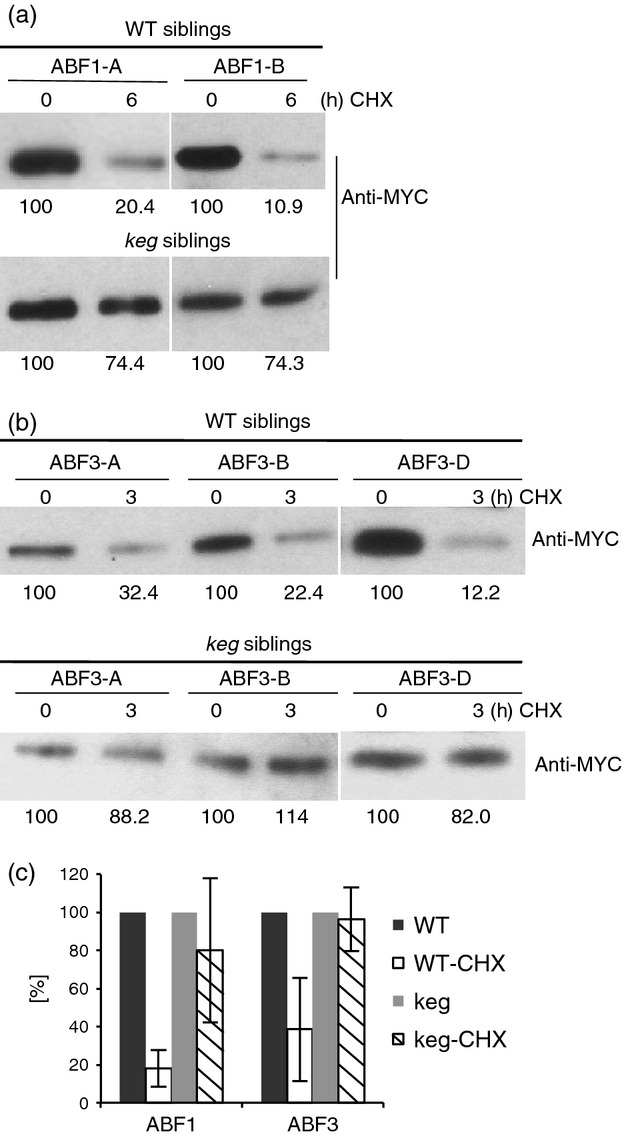
ABF1 and ABF3 protein degradation is slowed in *keg*.Seedlings from independent transgenic lines in *+/keg* background expressing Myc-ABF1 (a) or Myc-ABF3 (b) under the control of the *35S* promoter were grown for 6 days [wild-type (WT) siblings] or 14 days (*keg* siblings) and treated with cycloheximide for the indicated times. CHX 0 represents mock treatment with solvent alone. Proteins were extracted and immunoprecipitated by anti-Myc beads. Results were visualized by anti-Myc immunoblotting. The percentages represent western blot signal intensity quantified by ImageJ and compared with CHX 0.(c) Data from three independent experiments were plotted normalized to CHX 0 (ABF1, *n* = 6; ABF3, *n* = 8). The percentage remaining is statistically different in *keg* samples than in the WT by a Student's *t*–test: *P* < 0.0031 and *P* < 0.0002, for ABF1 and ABF3, respectively.

For this reason, we measured degradation rates directly with CHX chase assays in *keg* and wild-type sibling seedlings (Figure 4). In the wild type, more than 80% of Myc-ABF1 was degraded after 6 h of CHX treatment, whereas only 26% of Myc-ABF1 was degraded in *keg* seedlings (Figure 4a). Similarly, more than 68% of Myc-ABF3 was degraded after 3 h of CHX treatment in the wild type, whereas less than 20% of Myc-ABF3 was degraded in *keg* seedlings (Figure 4b). Combining all experiments, these results show that the *in vivo* protein degradation of both ABF1 and ABF3 is slower in *keg* (Figure 4c).

### The degradation of ABF1 and ABF3 is also slowed in *keg* extracts

We investigated the degradation of recombinant ABF1 and ABF3 in cell-free degradation assays (Wang *et al*., 2009). We tested whether ABF1 and ABF3 are degraded in a proteasome-dependent manner *in vitro* in wild-type seedling extracts, as reported for RGA1 (Wang *et al*., 2009). His-Flag-ABF1 or His-Flag-ABF3 was incubated in extracts from wild-type seedlings with or without proteasome inhibitor MG132. Without MG132, His-Flag-ABF1 and His-Flag-ABF3 were degraded rapidly, with most of the protein degraded after 90 min, whereas MG132-treated extracts had little or no detectable protein loss (Figure S3a). To further support the hypothesis that the ubiquitin pathway is active *in vitro*, we determined whether *in vitro* degradation depends on ATP, which is required for ubiquitylation and proteasome activity. Protein extracts without the addition of ATP were treated with apyrase, an ATPase. Similar to MG132, apyrase largely reduced the degradation of His-Flag-ABF1 and His- Flag-ABF3 (Figure S3b). The effects of MG132 and apyrase on *in vitro* degradation suggest that recombinant ABF1 and ABF3 protein degradation is proteasome dependent. Bacterially expressed ABFs with a different epitope tag, hemagglutinin (HA), were degraded *in vitro* in an identical manner, indicating that their behavior is independent of the epitope tag (Figure S4); however, the *in vitro* degradation assay could not recapitulate the ABA regulation of ABF proteolysis observed in transgenic seedlings (Figure S5).

**Figure 5:**
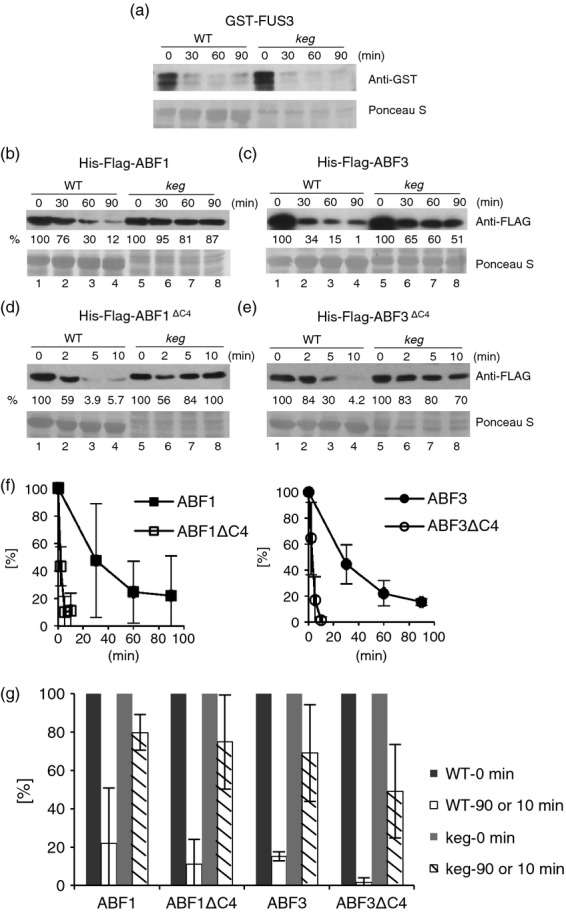
*In vitro* degradation of ABF1 and ABF3 is slowed in *keg*.Bacterially expressed recombinant GST-FUS3 (a), His-Flag-ABF1 (b), His-Flag-ABF3 (c), His-Flag-ABF1^ΔC4^ (d) or His-Flag-ABF3^ΔC4^ (e) was incubated with a 7–day-old Col or a 14–day-old *keg* seedling extract over the indicated time courses [note the shorter times in (d) and (e) compared with (b) and (c)]. His-Flag-tagged protein levels were visualized by anti-Flag immunoblotting. Ponceau S staining was used as the loading control. (f) Data from independent experiments comparing the *in vitro* degradation of full-length proteins and their respective ΔC4 forms were plotted normalized to time 0 (ABF1, *n* = 3; ABF3, *n* = 2).(g) Protein at 90 or 10 min for full-length and ΔC4 forms, respectively, were normalized to time 0. Student's *t*–tests indicate that the loss of the same protein was significantly slower in *keg* extracts compared with the wild type (WT; *n* = 3; *P* < 0.03, *P* < 0.02 and *P* < 0.03 for ABF1, ABF1^ΔC4^ and ABF3 ^ΔC4^, respectively). (Test not performed for ABF3, this specific time course was repeated twice, and a different time course, with the same results was performed once.) Note: cannot compare full-length and ΔC4 forms here because time points are different (see Figures 5f and 6 for these comparisons).

We next used the cell-free degradation assay to determine whether ABF1 or ABF3 degradation is slowed in *keg* extracts *in vitro*, as previously observed *in vivo*. The equivalent *in vitro* proteolysis of GST-FUSCA3, a B3 domain-containing transcription factor involved in embryogenesis previously demonstrated to be unstable (Lu *et al*., 2010), in wild-type and *keg* extracts demonstrates active proteolysis in *keg* extracts (Figure 5a). In wild-type protein extracts, 12% of His-Flag-ABF1 (Figure 5b, lane 4) or 2% of His-Flag-ABF3 (Figure 5c, lane 4) remained after 90 or 20 min, respectively, whereas 87 and 87%, of His-Flag-ABF1 and His-Flag-ABF3, respectively, remained in *keg* extracts (Figure 5b,c, lanes 8). The slower *in vivo* (Figure 4) and *in vitro* (Figure 5) degradation of tagged ABF1 and ABF3 in *keg* indicates ABF1 and ABF3 degradation is modulated by KEG E3 ligase.

### Deletion of the C4 domain destabilizes ABF1 and ABF3

A proteomic study in barley leaves showed most ABI5-related bZIP transcription factors have a conserved 14–3–3 binding motif (RRTLTGPW, within the C4 domain) at their C termini (Schoonheim *et al*., 2007a,b). To determine whether the C4 domain affects ABF1 and ABF3 degradation, we expressed recombinant ABF1 and ABF3 lacking the C4 domain (ΔC4), and monitored their degradation in the cell-free assay.

**Figure 6:**
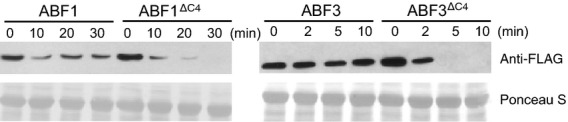
The deletion of nine C–terminal amino acids in the C4 domain destabilizes ABF1 and ABF3. Bacterially expressed recombinant His-Flag-ABF1 or His-Flag-ABF3 was incubated with 7–day-old Col seedling protein extract over the indicated time course. His-Flag-tagged protein levels were visualized by anti-Flag immunoblotting. Ponceau S staining was used as the loading control.

Similar to the full-length proteins, *in vitro* proteolysis of both ΔC4 forms was sensitive to MG132 and apyrase (Figure S6). When degradation time courses of the full-length and ΔC4 were compared directly, the ΔC4 forms were degraded more rapidly (Figures 5b–f and 6). HA-tagged forms of the same proteins showed the same trend, with the ΔC4 forms degraded more rapidly (Figure S7), indicating the effect is independent of the epitope tag. Thus the removal of the C4 domain destabilized both ABF1 and ABF3 *in vitro*.

Similar to full-length ABF1 and ABF3, *in vitro* proteolysis of His-Flag-ABF1^ΔC4^ and His-Flag-ABF3^ΔC4^ was slowed in *keg*. Less than 6% of His-Flag-ABF1^ΔC4^ and His-Flag-ABF3^ΔC4^ proteins remained after 10 min in wild-type extracts (Figure 5d,e, lanes 4, respectively), whereas all of His-Flag-ABF1^ΔC4^ and 70% of His-Flag-ABF3 ^ΔC4^ remained in *keg* extracts (Figure 5d,e, lanes 8, respectively). After combining all experiments, analysis demonstrates that protein loss is significantly slower in *keg* seedlings compared with the wild type (Figure 5g).

### ABF1 and ABF3 protein levels are affected by proteasomal inhibition, but not by ABA in *keg*

**Figure 7:**
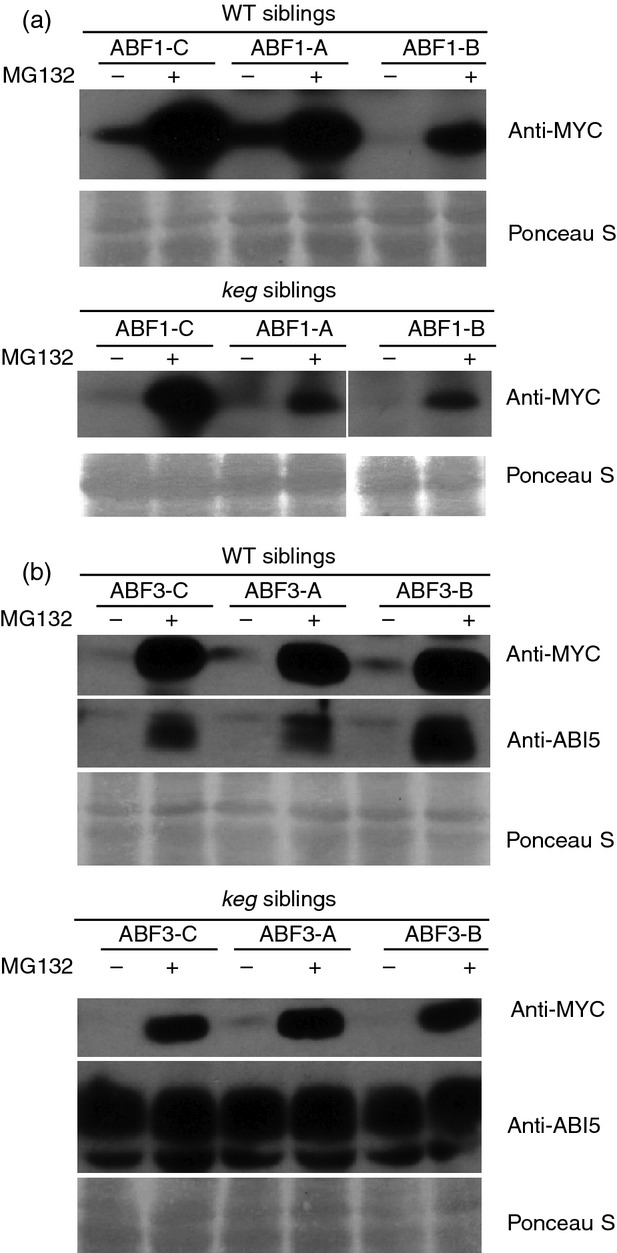
MG132 treatments result in ABF accumulation in *keg*. Seedlings from independent transgenic lines in the *+/keg* background expressing Myc-ABF1 (a) or Myc-ABF3 (b) in the *KEG/keg* background were grown under constant light for 14 days and treated with MG132, or DMSO as a mock treatment, for 6 h. Proteins were extracted and the results were visualized by anti-Myc and anti-ABI5 immunoblotting (b). Ponceau S staining was used as the loading control.

Because Myc-ABF1 and Myc-ABF3 degradation was slowed but not eliminated in *keg* seedlings (Figure 4), we asked whether the *keg*-independent degradation required the proteasome and was affected by ABA. Both Myc-ABF1 and Myc-ABF3 proteins accumulated in *keg* seedlings after 6 h of MG132 treatment, indicating Myc-ABF1 and Myc-ABF3 *keg*-independent degradation is proteasome dependent (Figure 7a,b, *keg* seedlings immunoblot). In contrast, endogenous ABI5 levels were not affected by proteasome treatment in *keg* seedlings, in contrast to the strong increase observed in the wild type, as shown in the Myc-ABF3 lines (Figure 7b).

**Figure 8:**
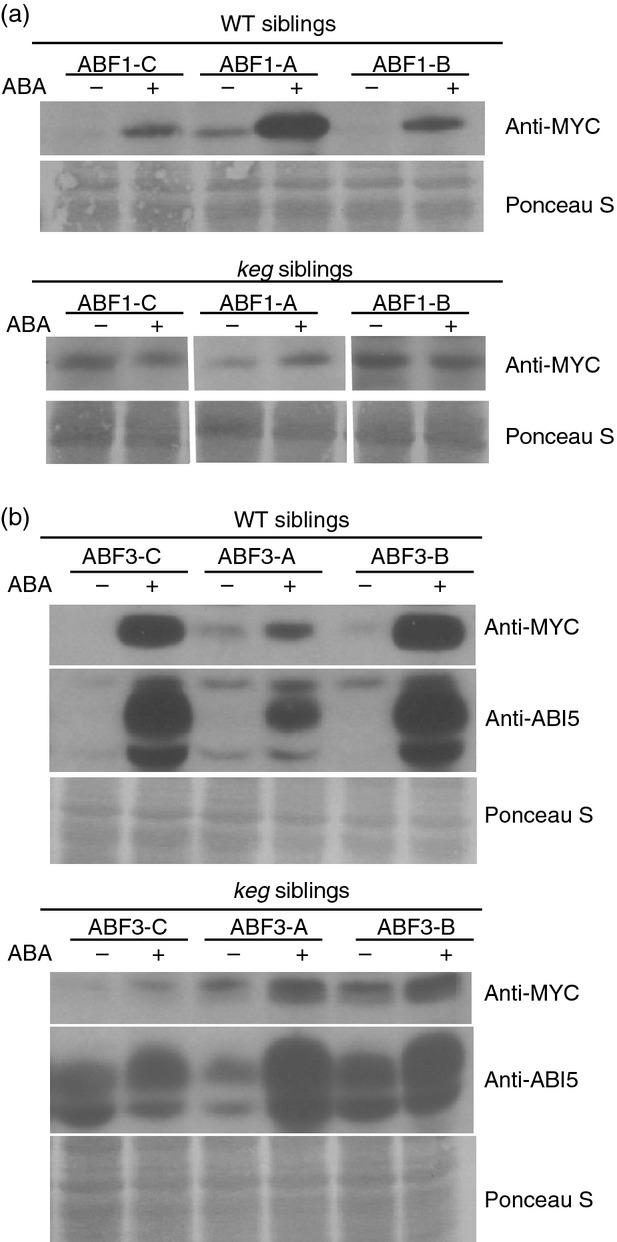
ABF1 and ABF3 protein levels are affected by ABA in the wild type (WT) but not in *keg* seedlings. Seedlings from independent transgenic lines in the *+/keg* background expressing Myc-ABF1 (a) or Myc-ABF3 (b) were grown under constant light for 14 days and treated with ABA, or ethanol as a mock treatment, for 6 h. Proteins were extracted and the results were visualized by anti-Myc or anti-ABI5 immunoblotting (b). Ponceau S staining was used as the loading control.

Wild-type or *keg* seedlings expressing Myc-ABF1 or Myc-ABF3 were also treated with ABA. Myc-ABF1 increased after 6 h of ABA treatment in the wild type, as observed previously (Figure 2), but this was not consistently the case in *keg* seedlings (Figure 8a). For Myc-ABF3, transgenic lines A and B accumulated slightly more Myc-ABF3 protein in ABA-treated *keg* seedlings compared with control *keg* seedlings, but two other lines did not (line C, Figure 8b, and line D, not shown). Endogenous ABI5 strongly accumulated with ABA treatment in wild-type seedlings, indicating that the ABA response was functional in these seedlings at this developmental stage. In *keg* seedlings, ABI5 levels were high without ABA, and increased slightly with ABA (Figure 8b).

### KEG is capable of ubiquitylating ABF1 and ABF3 and their C4 deletion forms *in vitro*

**Figure 9:**
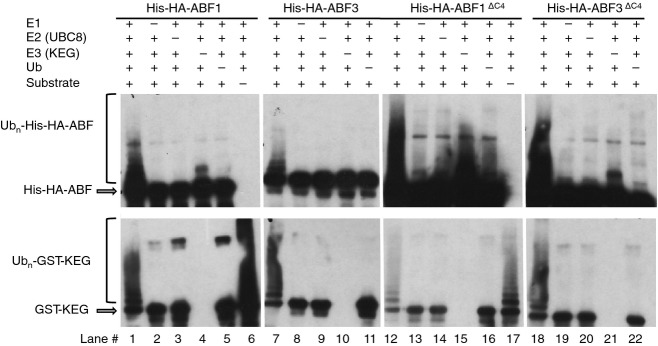
KEG ubiquitylates ABF1 and ABF3 and their C4 deletion forms *in vitro*. *In vitro* ubiquitylation assays with yeast E1, *Arabidopsis* His-UBC8 (E2), GST-KEG-RK (E3), ubiquitin (Ub), and His-HA-ABF1, His-HA-ABF3 or their C4 deletion forms (substrates). Substrate proteins visualized by anti-HA immunoblot (top). GST-KEG self-ubiquitylating E3 activity indicated by higher migrating forms in the anti-GST immunoblot (bottom).

The CHX chase and cell-free degradation assays demonstrated that ABF1 and ABF3 protein degradation is affected by the loss of KEG. To determine whether KEG acts directly through catalyzing ABF ubiquitylation, we performed *in vitro* ubiquitylation assays using recombinant KEG (GST-KEG), as the E3 ligase E3 and with ABF proteins as substrates. In complete ubiquitylation reactions, higher molecular mass forms of full-length His-HA-ABF1 and His-HA-ABF3, and their ΔC4 forms, were detected by anti-HA antibody (Figure 9, top panel, lanes 1, 7, 12 and 18). GST-KEG activity in these assays can be verified by self-ubiquitylation (Figure 9, lower panels, lanes 1, 6, 7, 12, 17 and 18). Omitting any of the required components abolished higher molecular mass forms, demonstrating that these forms are ubiquitylated by KEG (Figure 9).

### KEG interacts with ABF1, ABF3 and their C4 deletion forms *in vitro*

To further verify that KEG directly interacts with ABF1 and ABF3, we performed *in vitro* GST pull-down assays with KEG. Recombinant His-HA-ABF1, His-HA-ABF3 or ΔC4 forms were incubated with different forms of KEG. All bead-bound GST-KEGs, including the one used for *in vitro* ubiquitylation assays, pulled down all of the proteins tested (Figure S8).

### Loss of ABF1 or ABF3, or both, only partially rescues the *keg* phenotype

Previous studies indicated that ABI5 hyperaccumulation in *keg* contributes in part to the *keg* phenotype (Stone *et al*., 2006); however, because loss of *ABI5* does not fully rescue the *keg* phenotype, we hypothesized that hyperaccumulation of other KEG substrates, such as ABF1 and ABF3, could contribute to the *keg* phenotype. To test this hypothesis, we introduced *abf1* and *abf3* T–DNA insertional mutant alleles into *KEG/keg–1* mutant and *KEG/keg–1 abi5–1* mutants, and compared the phenotypes of *abf keg* seedlings compared with *keg* alone. Wild-type siblings (*KEG/KEG* or *KEG/keg* background) did not show any phenotypic differences among all the mutants. When compared with *keg* seedlings, *abf1 keg*, *abf3 keg*, and *abi5 keg* double mutants showed slightly better growth, with the emergence of the first pair of true leaves, not seen on *keg* seedlings (Figure S9). *abf1 abi5 keg* and *abf3 abi5 keg* triple mutant seedlings, and *abf1 abf3 abi5 keg* quadruple mutant seedlings, also showed better growth when compared with *keg* seedlings, but there was no significant enhancement compared with the *abf1 keg* or *abf3 keg* mutants (Figure S9).

**Figure 10:**
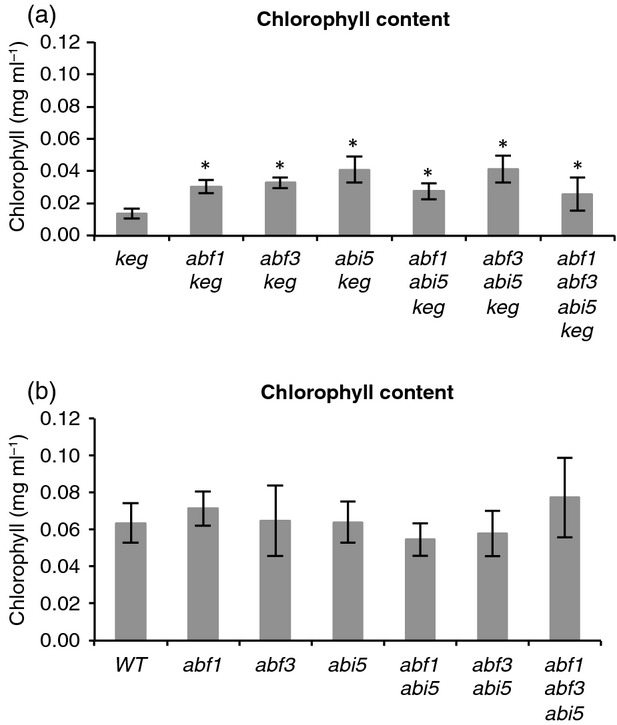
The loss of ABF1 or ABF3 only marginally rescues the *keg* phenotype.(a) Chlorophyll content of 10–day-old light-grown *keg* seedlings compared with *keg* seedlings with loss-of-function mutations in one or more *ABF* gene or *ABI5*.(b) Chlorophyll content of 10–day-old light-grown *KEG/KEG* or *KEG/keg* seedlings with loss-of-function mutations in one or more *ABF* gene or *ABI5*.Comparisons were made using anova with Tukey–Kramer *post-hoc* tests: *P < 0.007, compared with *keg* alone. Results are shown as means ± SDs for three biological replicates (*n* = 9). All *KEG/KEG* or *KEG/keg* seedlings had significantly more chlorophyll than their corresponding *keg* siblings (anova, *P* < 0.05).

Consistent with more visible greening, the chlorophyll content of 10–day-old light-grown seedlings was significantly higher in double, triple and quadruple mutants, compared with *keg* alone, but there were no significant differences among those mutants (Figure 10a), again indicating no additional rescue with loss of additional ABF proteins. Compared with their wild-type siblings, all *keg* seedlings have much lower chlorophyll levels and there are no statistically significant differences among the *abi5/abf* mutants in *KEG* seedlings (Figure 10b).

## Discussion

ABF1 and ABF3 are two ABFs with similar functions to ABI5 in regulating seed germination and post-germinative growth. ABI5, ABF1 and ABF3 are able to interact with ABI3, a transcription factor involved in seed maturation and dormancy, and ABF3 is functionally redundant to ABI5 in regulating ABA-mediated growth inhibition in seedlings (Finkelstein *et al*., 2005).

The *in vivo* degradation of Myc-tagged ABF1 and ABF3 showed that ABF1 and ABF3 proteins are unstable *in vivo*. ABF1 and ABF3 protein accumulated in the presence of the proteasome inhibitor MG132. The *in vivo* results were further supported by *in vitro* degradation assays. Because the ubiquitin/proteasome system is an ATP-dependent pathway (Smalle and Vierstra, 2004), stabilization of ABF1 and ABF3 by MG132 and ATP depletion *in vitro* strongly suggests that the degradation of ABF1 and ABF3 is dependent on the ubiquitin/proteasome system.

Treatment with ABA results in increases in transcripts of the ABI5-related bZIP transcription factors (Choi *et al*., 2000; Uno *et al*., 2000; Kim *et al*., 2002; Fujita *et al*., 2005). ABI5 protein is also affected by ABA (Lopez-Molina *et al*., 2001). Sirichandra *et al*. (2010) showed that YFP-ABF3 protein increased in response to ABA in 3–week-old transgenic plants. Consistent with their observation, we found that ABF1 and ABF3 proteins accumulated in response to ABA in 6–day-old seedlings. Moreover, we measured the *in vivo* degradation rates directly. ABF1 and ABF3 degradation rates are slowed in the presence of exogenous ABA, indicating that the accumulation with ABA likely results from changes in protein degradation.

The conserved C–terminal RRTLTGPW motif among ABFs is important for the 14–3–3 interaction. In a yeast two-hybrid (Y2H) assay, *Hordeum vulgare* (barley) ABI5-related bZIP transcription factors HvABF1, HvABF2 and HvABF3 interacted with the five barley 14–3–3 isoforms (with one exception), but not when the motif was deleted (Schoonheim *et al*., 2007a,b). In Arabidopsis, *in vitro* interaction between ABF3 and 14–3–3 is mediated by OST1 phosphorylation of threonine 451 in the C4 domain (Sirichandra *et al*., 2010). Because phosphorylation of Thr451 is also important for ABA-mediated ABF3 protein accumulation, Sirichandra *et al*. (2010) proposed that the binding of 14–3–3 to the phosphorylated C4 domain protects ABF3 from rapid protein degradation. Our *in vitro* degradation results with C4 deletion forms support this hypothesis. Deletion of the C4 domain accelerates ABF1 and ABF3 degradation *in vitro*. Because *in vitro* degradation of ABF1 and ABF3 C4 deletion forms is greatly slowed by MG132 and apyrase, the rapid degradation of these forms is still mediated by the ubiquitin/proteasome system. These results suggest that without the C4 domain, ABF1 and ABF3 cannot bind 14–3–3 proteins, and therefore lose some protection from degradation. Therefore, a conserved mechanism of stabilizing ABF1 and ABF3 proteins by ABA could be achieved by promoting phosphorylation at the conserved motifs, possibly by the SnRK2 kinases, which then results in increased interaction with 14–3–3 proteins.

Consistent with the model that ABI5 degradation is modulated by the E3 ligase KEG, loss of KEG results in ABI5 protein hyperaccumulation (Stone *et al*., 2006). Similarly, if ABF1 and ABF3 proteolysis requires KEG, the loss of KEG should confer ABF1 and ABF3 hyperaccumulation. We were unable to obtain antibodies against these proteins; therefore, we introduced transgenes expressing epitope-tagged forms under the control of a constitutive promoter. Myc-tagged ABF1 and ABF3 do not accumulate as strongly as ABI5 in *keg* mutant seedlings, because for an unknown reason the mRNA levels for Myc-ABF3 are unequal between wild-type and *keg* seedlings. Thus, comparison of steady-state protein is an inappropriate method to determine whether the transcription factors are post-translationally modulated by KEG. Therefore, we examined Myc-ABF1 and Myc-ABF3 proteolytic rates directly in the wild type and in the *keg* mutant. The slower degradation of ABF1 and ABF3 in the *keg* mutant *in vivo* and *in vitro* strongly suggests that protein degradation of ABF1 and ABF3 is modulated by KEG. Because the phenotype of the *keg* mutant is extremely different from wild-type plants, one might argue that the slower *in vitro* protein degradation in the *keg* extract results from a lack of the ubiquitin/proteasome machinery. However, GST-fused FUS3 protein is degraded in *keg* extracts at a similar rate compared with the wild type, which indicates that *keg* extracts contain all the components required, and suggests that the slower degradation observed in *keg* is specific to ABF1 and ABF3.

Our *in vitro* binding assays and ubiquitylation assays provide more evidence to support our hypothesis that the degradation of ABF1 and ABF3 is at least in part directly modulated by KEG. KEG interacts with ABF1 and ABF3 in GST pull-down assays*,* and ubiquitylates ABF1 and ABF3 *in vitro*. Furthermore, we demonstrated that the ABF1 and ABF3 C4 domains are not required for interaction with KEG, and nor are they required for ubiquitylation by KEG, suggesting that other regions interact with KEG.

A previous study showed that the loss of *ABI5* in the *keg* background partially rescues the *keg* post-germinative growth inhibition phenotype (Stone *et al*., 2006). Here, we show that similarly, *ABF1* or *ABF3* loss slightly affects growth, but cannot fully rescue the *keg* phenotype. Surprisingly, the removal of both bZIP transcription factors in the *keg* or in the *keg abi5* background does not enhance the rescue. One explanation is that the expression of other ABI5-related bZIP transcription factors are increased when ABF1, ABF3 or ABI5 are absent and the proteins hyperaccumulate in *keg*, making bZIP transcription factor levels (albeit different members) comparable with those in *keg* single mutants. Cross regulation has been observed. *ABF3* mRNA is hyperinduced by ABA in *abi5*, and *ABI5* mRNA is hyperinduced in *abf3*. *ABF1* mRNA is also hyperinduced by ABA in *abf3*, *abi5* and especially in the *abf3 abi5* double mutant (Finkelstein *et al*., 2005). Because *keg* seedlings show ABA-mediated growth inhibition and ABA-hypersensitive phenotypes (Stone *et al*., 2006), it is plausible that other ABI5-related bZIP transcription factors are hyperinduced in the *abf1 abi5 keg* and *abf1 abi5 keg* triple mutants, and in the *abf1 abf3 abi5 keg* quadruple mutant. Alternatively, other unrelated, not yet identified proteins may hyperaccumulate in *keg* and arrest growth.

## Experimental Procedures

### Plant material, growth conditions and chlorophyll content

Seeds from *Arabidopsis thaliana* Col–0 ecotype (wild type) or transgenic lines in Col–0 ecotype were sterilized with 30% (v/v) bleach and 0.1% (v/v) Triton X–100 for 15 min, and then stratified in water at 4°C for 2 days, plated on growth media (GM; Zenser *et al*., 2003) and, if grown to maturity, transferred to soil and grown under constant light at 22°C in a growth chamber. The chlorophyll content of 10–day-old seedlings was determined as described by Arnon (1949). Genotypes of *abf1–1* (SALK_043079), *abi3–1* (SALK_075836), *abi5–1* (described in Finkelstein *et al*., 2005), and *keg* (described in Stone *et al*., 2006) alleles were identified by PCR (Table S1).

### Plasmid construction

ABF1 (At1g49720.1, TAIR10; note, not the representative gene model) and ABF3 (At4g34000.1) coding regions were amplified by PCR using cDNA from Col–0 seedlings with primers (Table S1) and recombined into pDONR201 according to the manufacturer's instructions (Gateway; Invitrogen, http://www.invitrogen.com). ABF1-pDONR201 (p9052) and ABF3-pDONR201 (p9053) were used as templates with primers (Table S1), and the PCR products were recombined with pDONR201, creating p9198 and p9196, the respective C4 deletion forms.

For generating transgenic plants expressing Myc-tagged ABF1 and ABF3, pDONR201 plasmids were recombined with pGWB21 vector (Nakagawa *et al*., 2007) by Gateway recombination. For expressing Flag-tagged recombinant proteins, pDONR201 plasmids were recombined with pEAK1, a plasmid modified from pDEST17 (Invitrogen) with Flag sequences inserted (Kraft, 2007). For expressing HA-tagged recombinant proteins, pDONR201 plasmids were used as templates for PCR (Table S1). PCR fragments were digested with *Ase*I or *Nde*I and *Bam*HI and ligated into a modified pET3c plasmid (p3782) with His-HA tag sequences at the N terminus. All clones were sequence verified. The isolation and cloning of *KEG* full-length and partial cDNAs were performed as previously described by Stone *et al*. (2006).

### Plant transformation and transgenic plant selection

Arabidopsis *KEG/keg–1* (Stone *et al*., 2006) plants (verified by PCR) were transformed by floral dip (Clough and Bent, 1998) with *Agrobacterium tumefaciens* strain AGL1. Plants homozygous for the *ABF* transgene in the T_2_ or T_3_ generation were used.

### Cycloheximide chase and immunoprecipitation

Seeds from *KEG/keg–1* parents expressing Myc-tagged proteins were germinated and grown on plates under constant light at 22°C for 4 days, and seedlings with the *keg* phenotype were transferred to liquid media and grown for nine additional days. Wild-type siblings and all others were grown and CHX assays performed as described previously (Dreher *et al*., 2006), with some modifications. Proteins were extracted by grinding frozen seedlings with plant immunoprecipitation (IP) buffer [50 mm Tris-HCl, pH 8.1, 150 mm NaCl, 0.5% (v/v) NP–40, 1 mm phenylmethylsulfonyl fluoride (PMSF), 50 μm MG132, and one Complete Protease Inhibitor Cocktail Tablet (Roche, http://www.roche.com)/10 ml] and 1 mg in a 500 μl volume was immunoprecipitated by 20 μl of anti-Myc beads (EZview™ Red Anti-c-Myc Affinity Gel; Sigma-Aldrich, http://www.sigmaaldrich.com) at 4°C. Beads were rinsed three times with IP buffer for 20 min, each at 4°C. Proteins were eluted by boiling beads in 50 μl of double-strength Laemmli Sample Buffer (LSB), and 20 μl eluates were separated by 8% SDS-PAGE and transferred to polyvinylidene difluoride (PVDF) membranes (Millipore, http://www.millipore.com). These experiments were repeated three times.

### Proteasome inhibitor and ABA treatments

Seeds from *KEG/keg–1* parents expressing Myc-tagged proteins were germinated and grown on plates under constant light at 22°C for 4 days. Wild-type and *keg* siblings were transferred to separate plates with liquid GM and grown for 10 days. All other plants were grown using the same conditions used for the CHX assays. Media were discarded and 1 ml of fresh media with either 50 μm MG132 [final 0.5% (v/v) DMSO] or with 0.5% (v/v) DMSO was added. For ABA treatment, media were discarded and 900 μl of fresh growth media were added. Subsequently, 100 μl of either 500 μm ABA (in 5% ethanol) or 100 μl of 5% (v/v) ethanol was added. After 6 h at 22°C under constant light, seedlings were processed as described above. Both MG132 and ABA treatment experiments were repeated three times for all lines.

### Recombinant protein purification

His-Flag-tagged or His-HA-tagged proteins were expressed in *Escherichia coli* strain BL21(DE3)pLysS or Lemo21(DE3). Cell pellets were resuspended in buffer [25 mm Tris-HCl, pH 7.5, 500 mm NaCl, 0.1% (v/v) Triton X–100, and one Complete Protease Inhibitor Cocktail Tablet (Roche) per 50 ml] and lysed by sonication. Ni-sepharose high-performance beads (GE Healthcare Life Sciences, http://www.gelifesciences.com) were added to cleared cell lysates and the mixture was incubated at 4°C for 1 h. Beads were washed three times at 4°C with buffer [25 mm Tris-HCl, pH 7.5, 300 mm NaCl, and 0.1% (v/v) Triton X–100]. Proteins were eluted with 25 mm Tris-HCl, pH 7.5, 150 mm NaCl, 0.01% Triton X–100 and 200 mm imidazole. His-tagged proteins were stored in the same buffer with 20% (v/v) glycerol at −80°C.

### *In vitro* degradation assay

Proteins were extracted from Col–0 or *keg* seedlings in degradation buffer, with or without ATP, as described by Wang *et al*. (2009). Protein concentrations were measured by Bradford protein assay (Sigma-Aldrich), and protein concentrations were made equivalent. For MG132 treatment, 50 μm MG132 or 0.5% DMSO as solvent control were added to reactions; for apyrase (Sigma-Aldrich) treatment, 15 U apyrase or 15 μl of sterile water as solvent control were added to the reactions. Samples were fractionated by SDS-PAGE and transferred to PVDF membranes. These experiments were repeated at least three times.

### Immunoblot analysis

For the anti-Myc immunoblot, membranes were incubated in blocking solution containing 5% (w/v) non-fat dry milk in TBS-tween (50 mm Tris-HCl, pH 7.5, 150 mm NaCl and 0.05% Tween 20) for 1 h, followed by 1 h of incubation with mouse anti-Myc monoclonal antibody horseradish peroxidase (HRP) conjugate (Roche) at a 1:1000 dilution. Membranes were then washed three times with TBS-tween for 15 min and visualized with ECL Plus Western Blotting Detection Reagents (GE Healthcare Life Sciences) or Pierce ECL Western Blotting Substrate (Thermo Scientific, http://www.thermoscientific.com). For anti-Flag and anti-HA blots, membranes were incubated in blocking solution for 30 min or 1 h, respectively, and incubated with anti-Flag (Sigma-Aldrich) or anti-HA antibody (Roche) at a 1:5000 dilution. Membranes were washed three times with TBS-tween for 15 min and visualized with SuperSignal West Pico Chemiluminescent Substrate (Thermo Scientific).

### RNA extraction and quantitative PCR

Seeds were germinated and grown in 1 ml of liquid media under constant light at 22°C for 6 days, and treated with 50 μm ABA or 0.5% ethanol for 6 h. Total RNA was isolated using the RNeasy Plant Mini Kit (Qiagen, http://www.qiagen.com). A 2.4–μg portion of total RNA was used in a final volume of 20 μl reverse transcription reaction, performed by using Superscript II reverse transcriptase (Invitrogen). Real-time PCR amplification was performed with 50 μl of reaction solution containing 1 μl of cDNA, 10 pmoles of primers (Table S1) and full-strength SYBR Green Master Mix (Applied Biosystems, http://www.appliedbiosystems.com). Relative transcript levels for mRNAs were obtained using the comparative cycle threshold (*C*_t_) method and normalized to *UBQ10* (cfx manager 2.1, Bio-Rad, http://www.bio-rad.com).

### *In vitro* ubiquitylation and GST pull-down assays

*In vitro* ubiquitylation assays using GST-KEG-RK were performed as previously described (Stone *et al*., 2006). For the pull-down assays, recombinant GST-KEG (versions described in Stone *et al*., 2006) or GST alone were bound to glutathione sepharose beads. About 150 ng of His-HA tagged ABFs were incubated with beads in binding buffer [50 mm Tris-HCl, pH 7.5, 250 mm NaCl, 0.5% (v/v) NP–40, 1 mm PMSF] at 4°C for 1 h. Beads were washed in binding buffer three times at 4°C. Proteins were eluted with elution buffer (25 mm Tris-HCl, pH 7.5, 150 mm NaCl, 0.01% Triton X–100 and 20 mm glutathione), and then analyzed by SDS-PAGE and immunoblotting. These experiments were repeated three times for each protein.

### Statistical analyses

All comparisons used the Student's *t*–test or anova with Tukey's *post-hoc* test for multiple comparisons via JMP (http://www.jmp.com). *P* values were reported in the text for each test, with significance defined as *P* < 0.05.

## SUPPORTING INFORMATION

**Figure S1.** Myc-ABF1 and Myc-ABF3 mRNAs do not change in response to ABA.

**Figure S2.** Myc-ABF3 does not hyperaccumulate in *keg* seedlings, probably because the level of transgenic mRNA is lower than in the wild type, whereas Myc-ABF1 slightly accumulates.

**Figure S3.** *In vitro* degradation of ABF1 and ABF3 is affected by MG132 and apyrase.

**Figure S4.** Recombinant ABF1 and ABF3 with HA tag can be degraded *in vitro*.

**Figure S5.** ABA pre-treatment of Col seedlings does not block ABF1 and ABF3 degradation *in vitro*.

**Figure S6.** *In vitro* degradation of ABF1 and ABF3 C4 deletions are slowed by MG132 and apyrase.

**Figure S7.** The deletion of nine amino acids in the C4 domain destabilizes ABF1 and ABF3 with an HA epitope tag.

**Figure S8.** KEG interacts with ABF1 and ABF3 *in vitro*.

**Figure S9.** Loss of *ABF1* or *ABF3* only partially rescues the *keg* phenotype.

**Table S1.** Primers for PCR and clone numbers.
