# Supplementary figures and images for: ABA and the ubiquitin E3 ligase KEEP ON GOING affect proteolysis of the Arabidopsis thaliana transcription factors ABF1 and ABF3
Source: Plant J. 2013 Jun 6;75(6):965–76. doi: 10.1111/tpj.12259 (PMC3823012; doi:10.1111/tpj.12259)

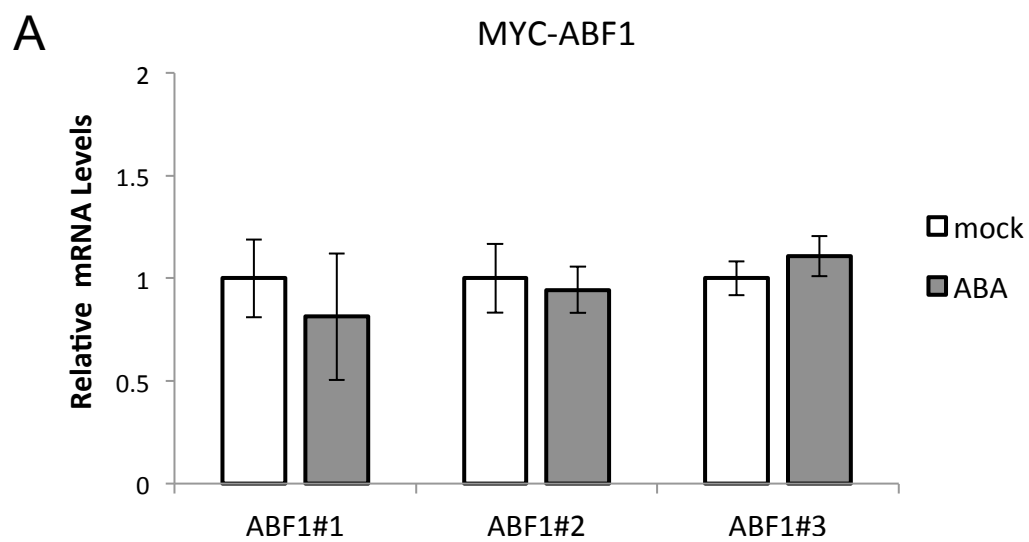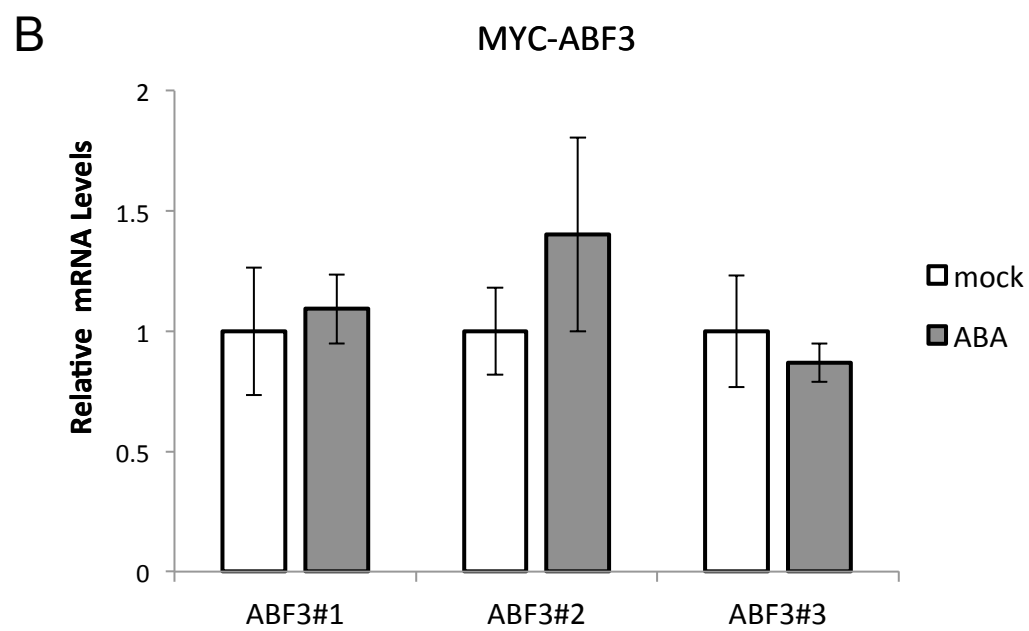

Figure S1.

Supplement: Supplementary file 1 [file tpj0075-0965-SD1.pdf]

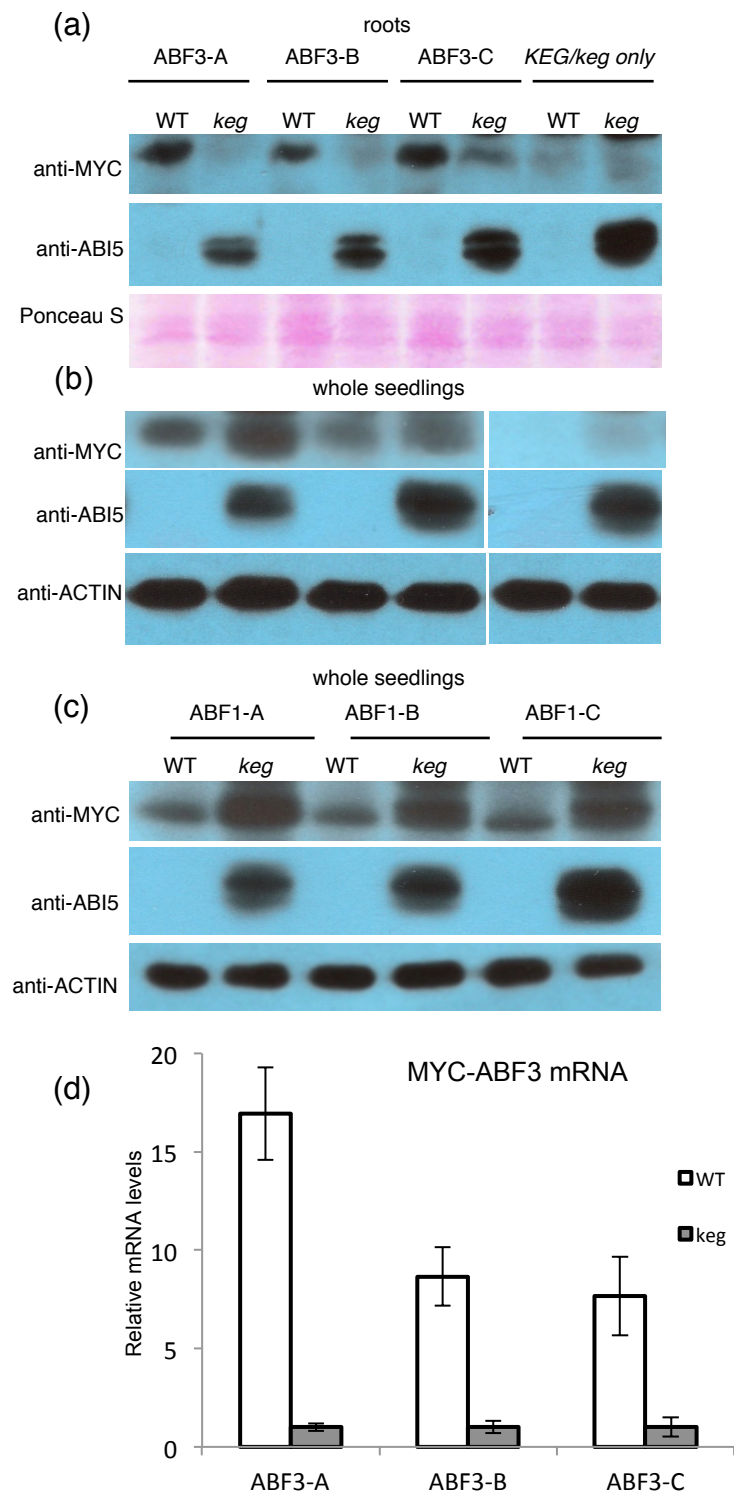

Figure S2.

Supplement: Supplementary file 2 [file tpj0075-0965-SD2.pdf]

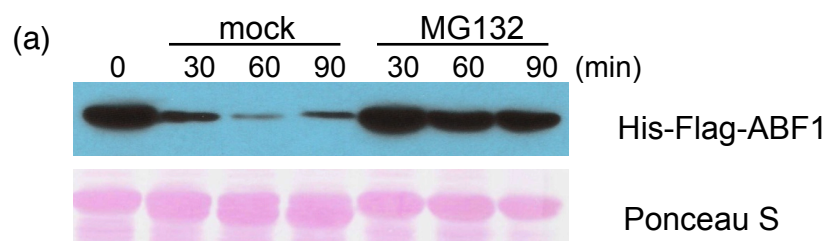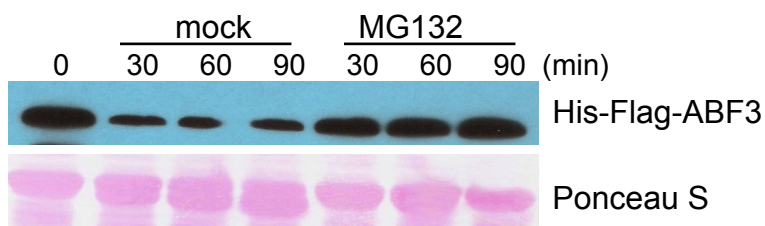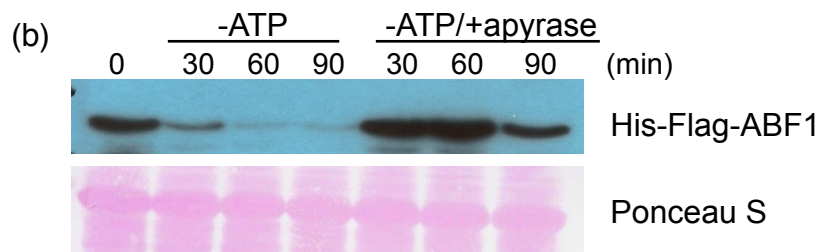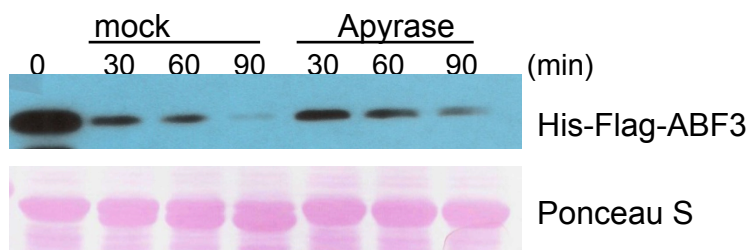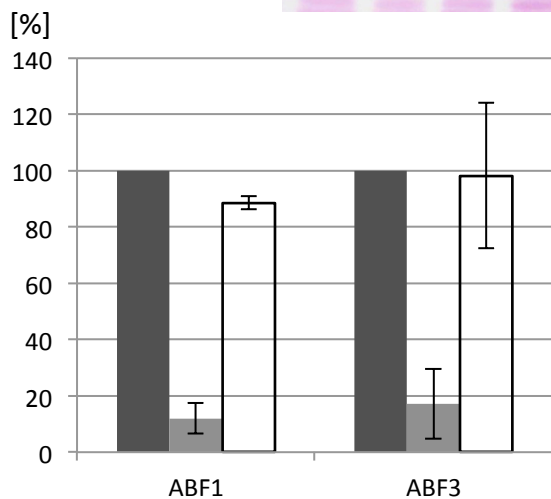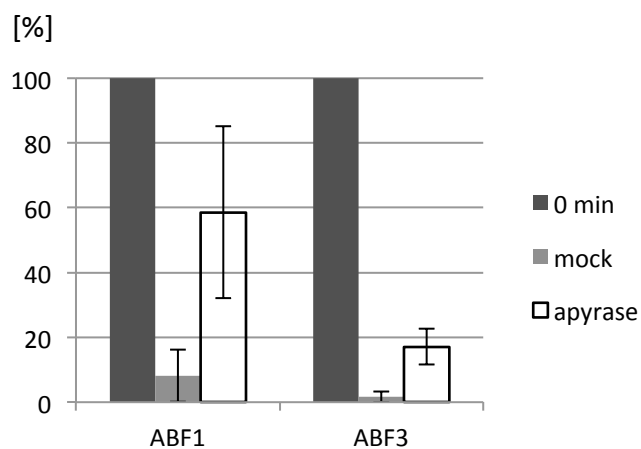

Figure S3.

Supplement: Supplementary file 3 [file tpj0075-0965-SD3.pdf]

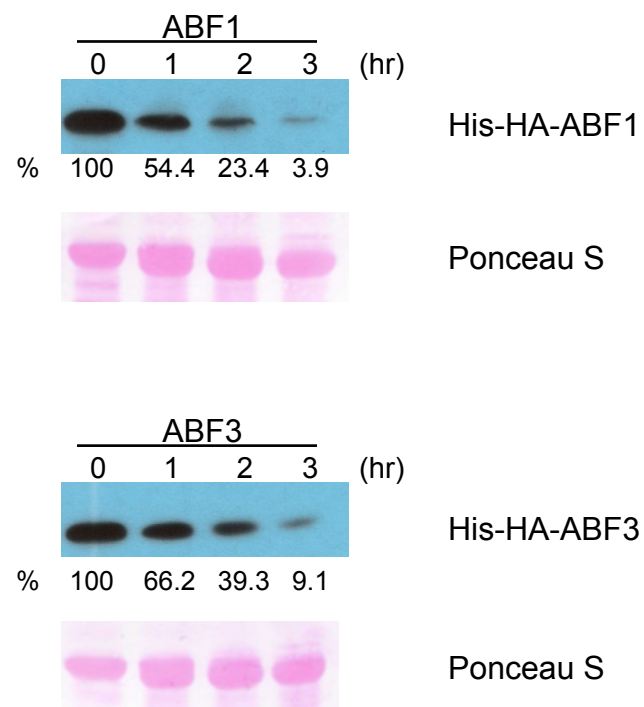

Figure S4.

Supplement: Supplementary file 4 [file tpj0075-0965-SD4.pdf]

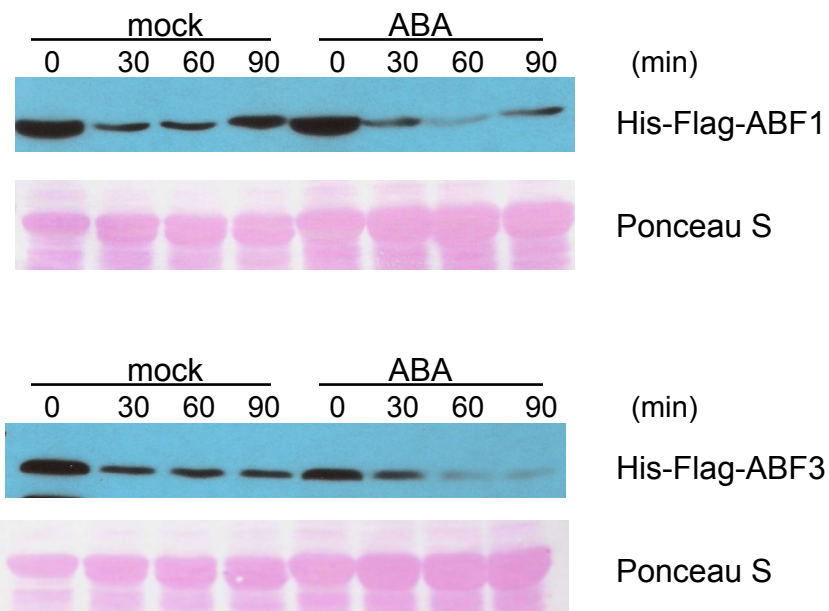

Figure S5.

Supplement: Supplementary file 5 [file tpj0075-0965-SD5.pdf]

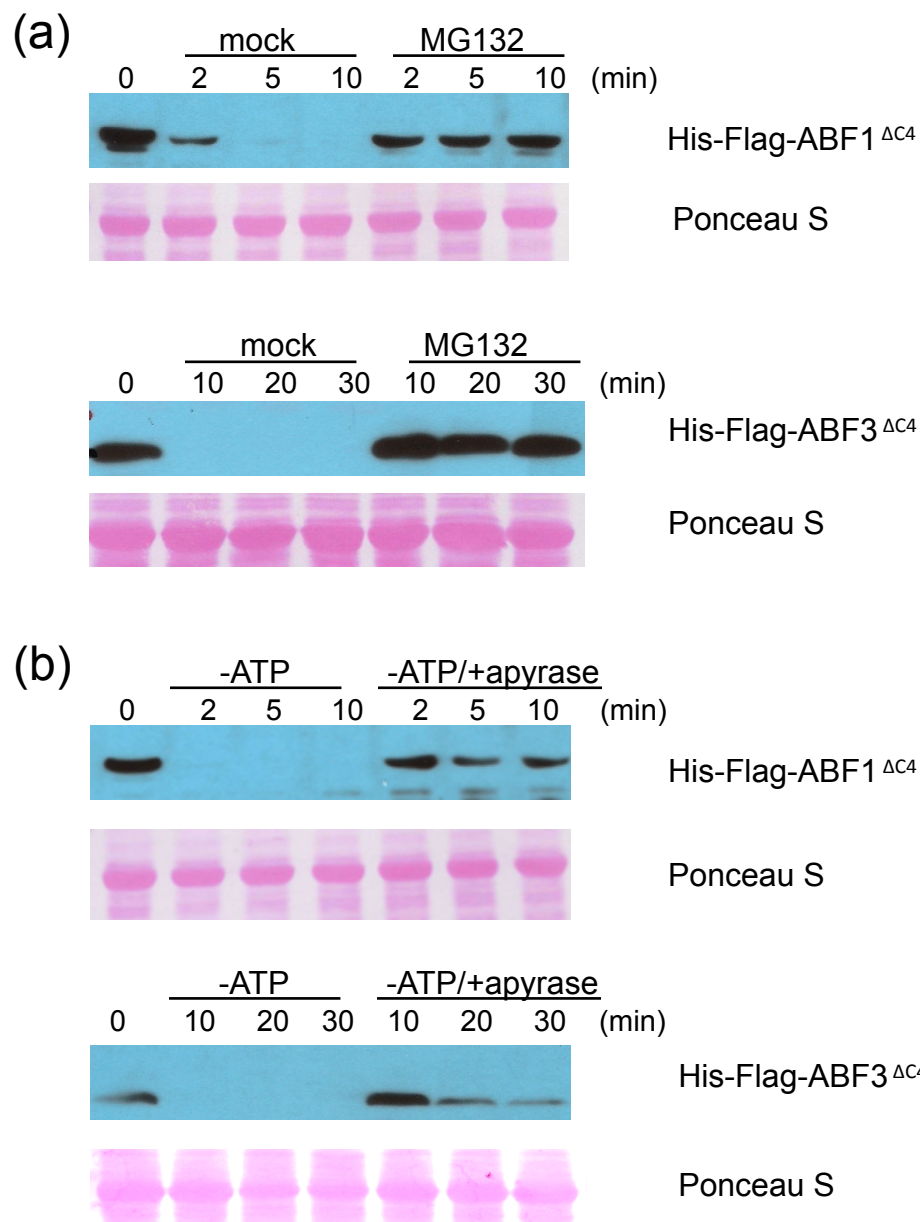

Figure S6.

Supplement: Supplementary file 6 [file tpj0075-0965-SD6.pdf]

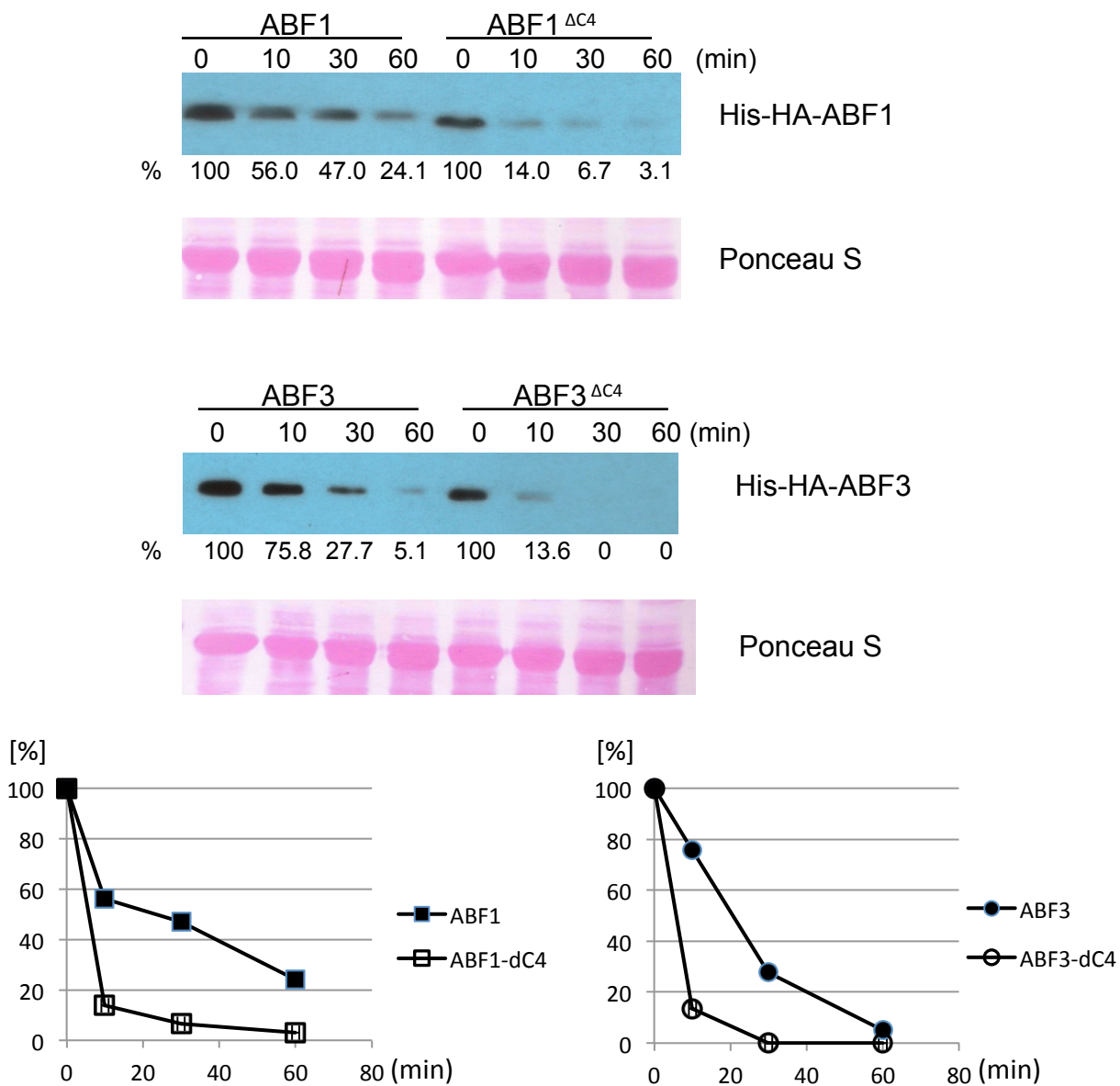

Figure S7.

Supplement: Supplementary file 7 [file tpj0075-0965-SD7.pdf]

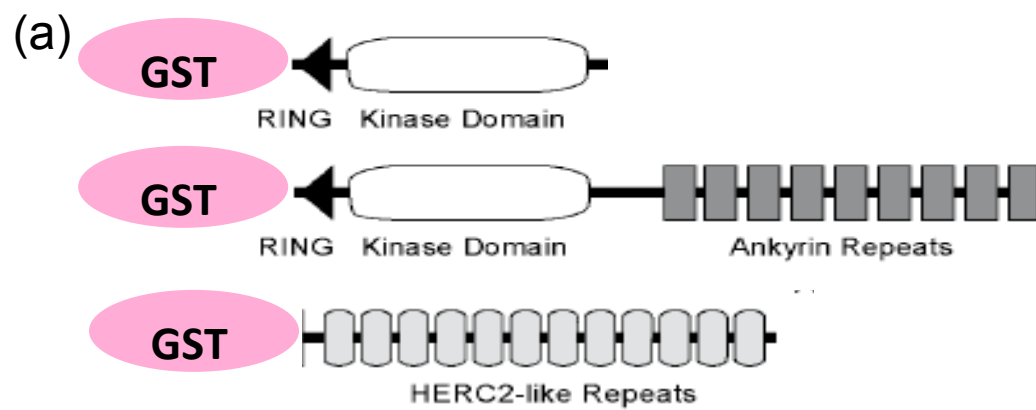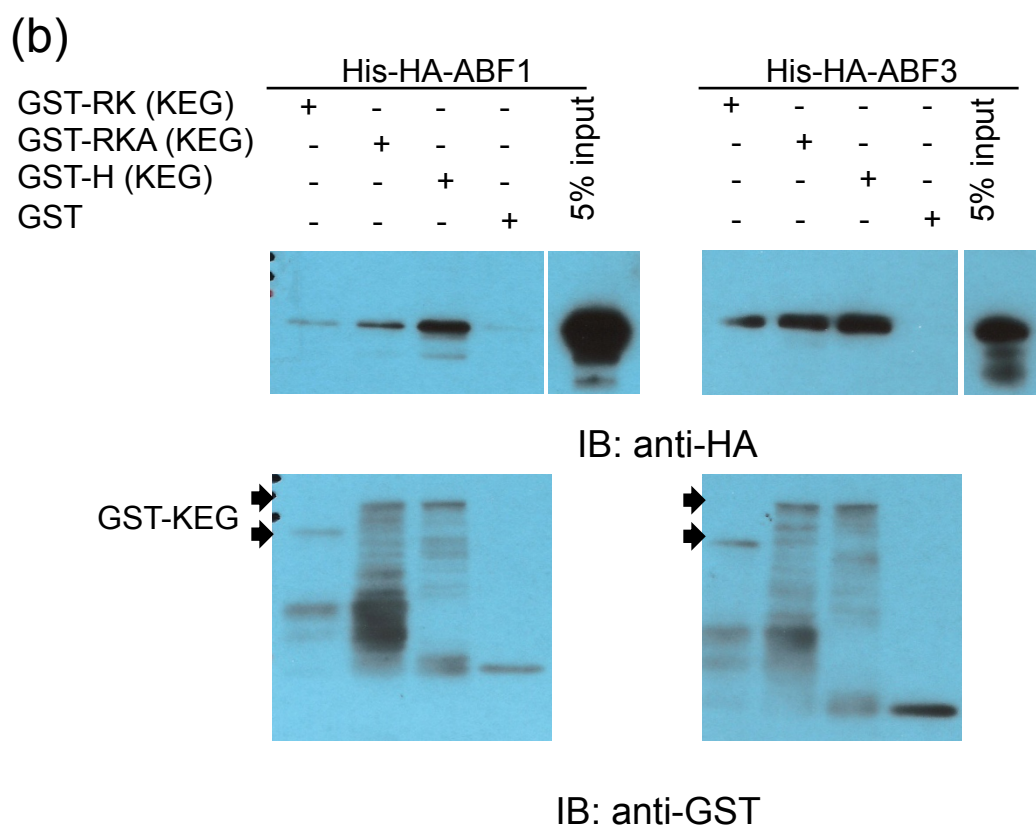

Figure S8.

Supplement: Supplementary file 8 [file tpj0075-0965-SD8.pdf]
