# Supplementary material for: ABA and the ubiquitin E3 ligase KEEP ON GOING affect proteolysis of the Arabidopsis thaliana transcription factors ABF1 and ABF3
Source: Plant J. 2013 Jun 6;75(6):965–76. doi: 10.1111/tpj.12259 (PMC3823012; doi:10.1111/tpj.12259)
Supplement: Supplementary file 9 [file tpj0075-0965-SD9.pdf]

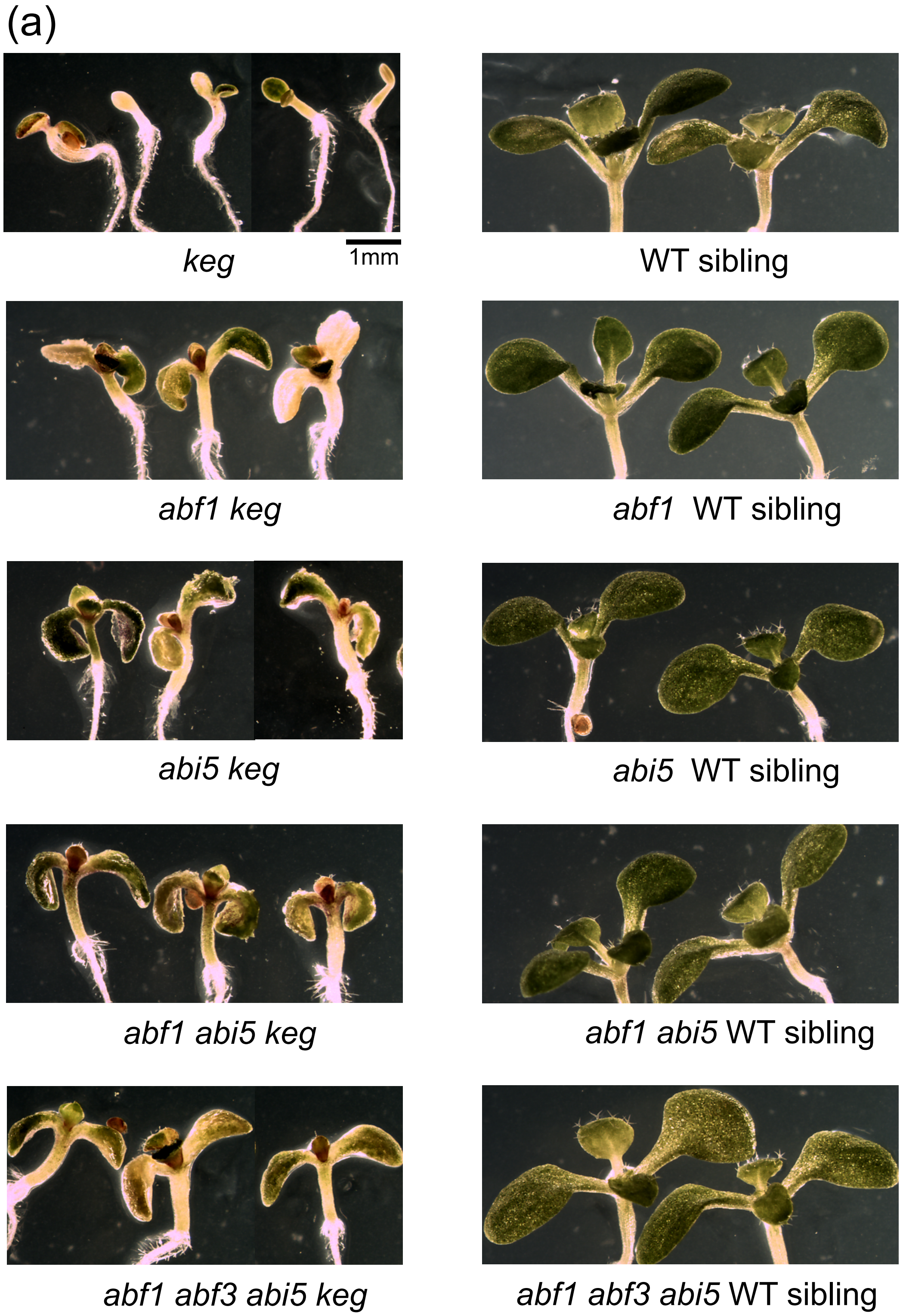

Figure S9, page 1.

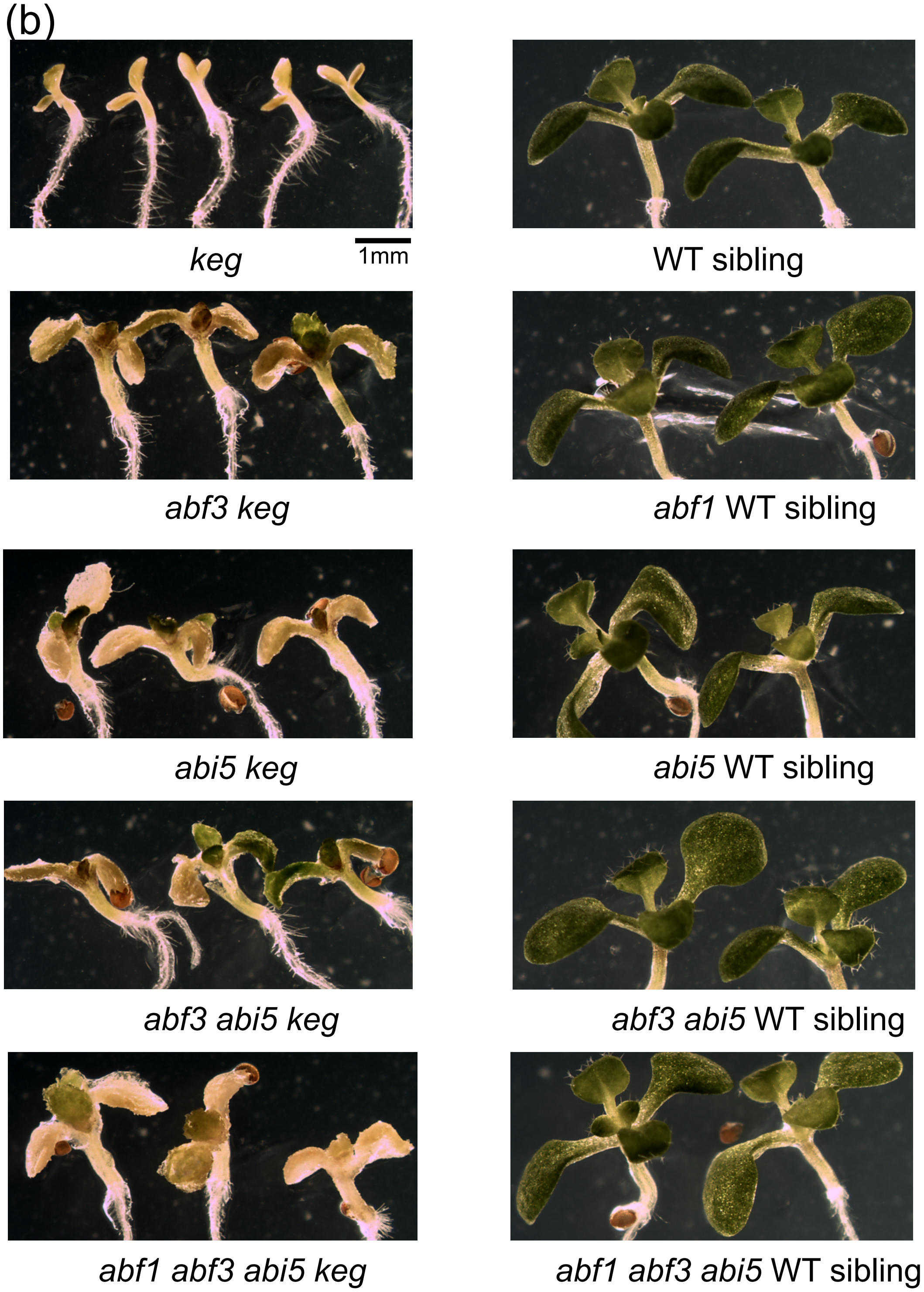

Figure S9, page 2.
