# Supplementary material for: ABA and the ubiquitin E3 ligase KEEP ON GOING affect proteolysis of the Arabidopsis thaliana transcription factors ABF1 and ABF3
Source: Plant J. 2013 Jun 6;75(6):965–76. doi: 10.1111/tpj.12259 (PMC3823012; doi:10.1111/tpj.12259)
Supplement: Supplementary file 10 [file tpj0075-0965-SD10.pdf]

primer number	5' or 3' (relative to ORF)	template	purpose	sequence (capital letters denote gene sequence)	plasmid	notes
9-325	5'	seedling cDNA	ABF1- pDONR201	5’-ggggacaagtttgtacaaaaaagcaggtcgATGGGTACTCACATTGATATCA-3’	p9052	
9-327	3'	seedling cDNA	ABF1- pDONR201	5’-ggggaccactttgtacaagaaagtgggtcTTACCACGGACCGGTAAGGG-3’	p9052	
9-328	5'	seedling cDNA	ABF3-pDONR201	5’- ggggacaagtttgtacaaaaaagcaggtcgATGGGGTCTAGATTAAACTTCA-3’	p9053	
9-330	3'	seedling cDNA	ABF3-pDONR201	5'-ggggaccactttgtacaagaaagctgggtc CTACCAGGGACCCGTCAAT-3’	p9053	
9-325	5'	p9052	ABF1-delta C4 pDONR201	5’-ggggacaagtttgtacaaaaaagcaggtcgATGGGTACTCACATTGATATCA-3’	p9198	
9-436	3'		ABF1-delta C4 pDONR201	5’-ggggaccactttgtacaagaaagtgggtcttaggccagcaatggaggctgctt-3’	p9198	
9-328	5'	p9053	ABF3-delta C4 pDONR201	5'-ggggaccactttgtacaagaaagctgggtcCTACCAGGGACCCGTCAAT-3’	p9196	
9-434	3'		ABF3-delta C4 pDONR201	5’-ggggaccactttgtacaagaaagtgggtcctagcatcccattccccatggctg-3’	p9196	
9-438	5'	p9052	His-HA-ABF1 into modpET3c	5’-gtcaattaatATGGGTACTCACATTGATAT-3’	p9200	
9-439	3'		His-HA-ABF1 into modpET3c	5’- gtcaggatccTTACCACGGACCGGTAAGG-3’	p9200	
9-442	5'	p9053	His-HA-ABF3 into modpET3c	5'-gtcacatATGGGGTCTAGATTAAACTTCAA-3’	p9202	
9-443	3'		His-HA-ABF3 into modpET3c	5’-gtcaggatccCTACCAGGGACCCGTCAATGT-3’	p9202	
9-438	5'	p9052	His-HA-ABF1deltaC4 into modpET3c	5’-gtcaattaatATGGGTACTCACATTGATAT-3’	p9201	
9-452	3'		His-HA-ABF1deltaC4 into modpET3c	5’- gtcaggatccTTAGGCCAGCAATGGAGGCTGCTT-3’	p9201	
9-442	5'	p9053	His-HA-ABF1deltaC4 into modpET3c	5'-gtcacatATGGGGTCTAGATTAAACTTCAA-3’	p9203	
9-453	3'		His-HA-ABF3deltaC4 into modpET3c	5’-gtcaggatccCTAGCATCCCATTCCCCATGGCTG-3’	p9203	
9-405	5'		UBQ10- for qPCR	5’-GGCCTTGTATAATCCCTGATGAA-3’		
9-406	3'		UBQ10- for qPCR	5’-AGAAGTTCGACTTGTCATTAGAAAGAAA-3’		
9-407	5'		Myc-ABF1- for qPCR	5’-TTGTACAAAAAAGCAGGCTCGAT-3’		
9-413	3'		Myc-ABF1- for qPCR	5’-GGCTTTGACTCATTCCCTCTAGAA-3’		
9-407	5'		Myc-ABF3- for qPCR	5’-TTGTACAAAAAAGCAGGCTCGAT-3’		
9-408	3'		Myc-ABF3- for qPCR	5-GCTGCTCACTCACACCATCAA-3’		
5-254			*KEG*	5’- GAGTGGATCCAGTGCACATC-3’		
9-001			*SALK T-DNA*	5-TGGTTCACGTAGTGGGCCATCG-3’		
5-483			*ABF1*	5'-GGTTTTCATTATTTCAGCCTGC-3'		SALK_043079
5-484			*ABF1* use with 9-001 (T-DNA)	5'–GGGACCTAGTGGTTTTGTTCC–3'		
5-491			*ABF3*	5'-TTTGTGTGACTGAGCTGCTTC-3'		SALK_075836
5-492			*ABF3* use with 9-001 (T-DNA)	5'-ACACCATTTCCAATGTCATCC-3'		
9-343			*ABI5*	5'-GGTTATTGTTGTGTATATGATGCAGTTG-3'		*abi5-1*
9-344			*ABI5*	5'-CCACTACTCTTTTCCTTCCCC-3'		*abi5-1*
