# Supplementary material for: ABA and the ubiquitin E3 ligase KEEP ON GOING affect proteolysis of the Arabidopsis thaliana transcription factors ABF1 and ABF3
Source: Plant J. 2013 Jun 6;75(6):965–76. doi: 10.1111/tpj.12259 (PMC3823012; doi:10.1111/tpj.12259)
Supplement: Supplementary file 11 [file tpj0075-0965-SD11.docx]

**Supplemental Figure Legends.**

**Figure S1. MYC-ABF1 and MYC-ABF3 transgene mRNAs do not change in response to ABA**

Transgenic seedlings expressing MYC-ABF1(a) or MYC-ABF3 (b) were grown for 6 days and treated with ABA or mock treated (solvent only) for 6 hours. Levels of transgene mRNA were assayed by quantitative RT-PCR. Results are shown as mean ± SE (n=6) normalized to *UBQ10* from three individual experiments. mRNA levels were not significantly different between ABA and mock treatment for all lines (Student’s t test, all p values > 0.2).

**Figure S2. Myc-ABF3 does not hyper-accumulate in keg seedlings likely because transgene mRNA is lower, while Myc-ABF1 slightly hyper-accumulates**

Myc-ABF3 levels in roots (a) and whole seedlings (b). Top, anti-myc IB, middle panel anti-ABI5 IB, Ponceau S stain for protein loading (a only), anti-actin IB for protein loading (b). (c) Myc-ABF3 levels in whole WT or keg seedlings. Panels as in (b). (d) Transgene mRNA as determined by qPCR. mRNAs are significantly higher in WT compared to keg by Student’s t test (p-values <0.0001, <0.0001 and <0.004, for ABF3A-C, respectively).

**Figure S3. *In vitro* degradation of ABF1 and ABF3 is regulated by MG132 and apyrase**

Bacterially expressed recombinant His-Flag-ABF1 or His-Flag-ABF3 was incubated with 7 day old Col seedling protein extract and with MG132 (a) or apyrase (b) or their respective mock treatments for the indicated times and graphed in (c) and (d), respectively. His-Flag tagged protein levels were visualized by anti-Flag immunoblotting. Ponceau S staining as loading control.

(c) Histograms of experimental data in (a) at 60 min; (d), histograms of data from (b) at 90 min. Values are statistically different from mock treatment using Student’s t test. For (c), ABF1 p-value <0.0001 (n=3), ABF3 p-value <0.008 (n=3). For (d), ABF1 and ABF3 p-value <0.01 (N=4 and 3, respectively).

**Figure S4. Recombinant ABF1 and ABF3 with HA tag can be degraded *in vitro***

Bacterially expressed recombinant His-HA-ABF1 or His-HA-ABF3 was incubated with 7 day old Col seedlings protein extract over indicated time course. His-HA tagged protein levels were visualized by anti-HA immunoblotting. The percentage numbers represent western blot signal intensity quantified by ImageJ and compared to time 0. Ponceau S staining as loading control.

**Figure S5. ABA pre-treatment of Col seedlings does not stop ABF1 and ABF3 *in vitro* degradation**

Bacterially expressed recombinant His-Flag-ABF1 or His-Flag-ABF3 was incubated with protein extracts from 7-day-old Col seedlings pretreated with ABA or ethanol as mock treatment. ABA was also included in the extraction buffer. His-Flag tagged protein levels were visualized by anti-Flag immunoblotting. Ponceau S staining as loading control.

**Figure S6. *In vitro* degradation of ABF1 and ABF3 C4 deletions are regulated by MG132 and apyrase**

Bacterially expressed recombinant His-Flag-ABF1 ^ΔC4^ or His-Flag-ABF3 ^ΔC4^ was incubated with 7-day-old Col seedling protein extract and with MG132 (a) or apyrase (b) over indicated time course. His-Flag tagged protein levels were visualized by anti-Flag immunoblotting. Ponceau S staining as loading control.

**Figure S7. Deletion of 9 amino acids in C4 domain destabilizes ABF1 and ABF3 with an HA epitope tag**

Bacterially expressed recombinant His-HA-ABF1 or His-HA-ABF3 was incubated with 7 day old Col seedlings protein extract over indicated time course. His-HA tagged protein levels were visualized by anti-HA immunoblotting. The percentage numbers represent western blot signal intensity quantified by ImageJ and compared to time 0. Ponceau S staining as loading control. This time course was performed twice with similar results.

**Figure S8. KEG interacts with ABF1 and ABF3 *in vitro***

(a) Diagram of truncations of KEG used in GST pull-down assays. GST tag is not to scale.

(b) Recombinant HIS-HA proteins were incubated with truncations of GST-KEG or GST bound to glutathione sepharose beads. Bead bound proteins were analyzed by anti-HA and anti-GST immunoblotting.

**Figure S9. Loss of ABF1 or ABF3 only partially rescues *keg* phenotype**

(a) 9 day old light-grown seedlings.

(b) 10 day old light-grown seedlings

The WT siblings are in *KEG/KEG* or *KEG/keg* background
